# Development and validation of a nomogram for predicting cough variant asthma diagnosis

**DOI:** 10.1186/s12890-025-03478-3

**Published:** 2025-01-20

**Authors:** Jiao Min, Xiaomiao Tang, Di Zhang, Jin Yang, Fei Li, Wei Lei

**Affiliations:** 1 Department of Pulmonary and Critical Care Medicine, The First Affiliated Hospital of Soochow University, Suzhou, Jiangsu 215006 China; 2 Department of Respiratory Medicine, The Second Affiliated Hospital of Hainan Medical University, 368 Yehai Avenue, Longhua District, Haikou, Hainan 570100 China

**Keywords:** Cough variant asthma (CVA), Chronic cough, Bronchial provocation test, Pulmonary function, Diagnostic prediction, Nomogram

## Abstract

**Background:**

Cough variant asthma (CVA) is a specific type of asthma characterized by chronic cough as the sole or predominant symptom. Accurate diagnosis is crucial for effective treatment, yet bronchial provocation test is not always feasible in clinical settings. To identify independent predictors of CVA diagnosis, we developed a nomogram for predicting CVA. Univariate and multivariate logistic regression analyses were employed to construct the model, and the accuracy and consistency of the prediction model were subsequently validated.

**Methods:**

We conducted a retrospective review of clinical data from 241 outpatients with chronic cough (≥ 8 weeks) who underwent bronchial provocation test at our hospital between January 2018 and December 2021. Patients were categorized into CVA group and Non-CVA group based on diagnostic criteria. Univariate analysis (chi-square and t-tests) was performed, followed by multivariate logistic regression to identify independent predictors. A nomogram was constructed using these predictors and validated using Bootstrap resampling (B = 200) to calculate the C-index. Additionally, receiver operating characteristic (ROC) curve analysis and decision curve analysis (DCA) were employed to assess the model's accuracy.

**Results:**

Of the 241 outpatients, 156 (64.7%) were diagnosed with CVA. Multivariate analysis identified several independent predictors of CVA, including cough triggered by cold air (OR = 12.493, *P* = 0.019), exposure to pungent odors (OR = 3.969, *P* = 0.002), cough phasing (OR = 4.515, *P* < 0.001), history of allergic rhinitis (OR = 3.231, *P* = 0.018), and the percentage of the predicted value of maximum mid-expiratory flow (MMEF%pred) (OR = 0.981, *P* = 0.039) were independent predictors of CVA. The nomogram demonstrated good discrimination (AUC = 0.829) and calibration, with a sensitivity of 75.3% and specificity of 77.6% at the optimal cutoff. The C-index was 0.920, indicating excellent model performance.

**Conclusions:**

We successfully developed and validated a user-friendly nomogram that accurately predicted CVA diagnosis based on clinical characteristics and pulmonary function test. This nomogram model could assist clinicians in diagnosing CVA, especially in patients without bronchial provocation test or with contraindications to bronchial provocation test.

Chronic cough is one of the most prevalent complaints among patients in general practice and respiratory specialist outpatient clinics, accounting for approximately 9.6% of the global adult prevalence [1–3]. Numerous studies have indicated that cough variant asthma (CVA) is a significant cause of chronic cough in adults, responsible for as much as 24% to 42% of cases [4, 5]. CVA is characterized by chronic cough as the sole or primary symptom, occurring in the absence of obvious wheezing or dyspnea; however, airway hyperresponsiveness is present, and anti-asthmatic treatment has been shown to be effective [6, 7]. This condition is typically marked by paroxysmal dry cough, which predominantly occurs at night or in the early morning and can be easily triggered by factors such as cold air or pungent odors [8–10].

While the bronchial provocation test is regarded as the gold standard for diagnosing CVA, its implementation necessitates specialized equipment, trained technicians, and stringent quality control, which can result in higher medical costs and present limitations, including examination risks and relative contraindications [11, 12]. At present, bronchial provocation test is not performed as widely as conventional lung function test [13]. Consequently, when patients are unable to undergo further examination to confirm the diagnosis, CVA is frequently overlooked or misdiagnosed, potentially leading to delayed treatment and progression to classic asthma [14, 15].

In recent years, clinical prediction models have emerged as valuable tools for estimating a patient's risk of developing specific diseases based on a combination of clinical variables. These models have demonstrated significant utility across various medical fields, including oncology and infectious diseases [16, 17]. Currently, several prediction models for asthma diagnosis have been developed by researchers [18–22], all of which exhibit strong predictive performance. However, these models primarily focus on typical asthma patients, revealing a notable gap in research regarding dedicated prediction models for CVA diagnosis.

Consequently, this study is the first to construct and validate a nomogram model aimed at predicting CVA diagnosis through a retrospective analysis of data from chronic cough patients who underwent bronchial provocation test. This model integrates readily available clinical and lung function parameters to create an easy-to-use nomogram by identifying independent predictors of CVA. By developing this CVA prediction model, we provide a feasible alternative diagnostic tool for chronic cough patients who require, but are unable to complete, the bronchial provocation test. This study aims to facilitate early diagnosis of CVA and inform clinical decision-making, ultimately enhancing patient prognosis.

## Materials and methods

### Study population and data

This study retrospectively analyzed the clinical data of 241 patients with chronic cough who received outpatient treatment in our hospital from 2018 to 2021. All included patients underwent a bronchial provocation test to determine the presence of CVA. Informed consent was obtained from each patient prior to the bronchial provocation test. The patient's basic information, including age, gender, smoking history, past medical history, basic pulmonary function test results, peripheral blood eosinophil count, and fractional exhaled nitric oxide (FeNO), was recorded in detail and included in the analysis. The study was approved by the ethics committee of the First Affiliated Hospital of Soochow University (No. 2021–215). It was a retrospective non-interventional study, and patients were exempt from informed consent.

### Inclusion and exclusion criteria

Inclusion criteria: 1) age ≥ 18 years old; 2) diagnosed with chronic cough, that is, cough symptoms lasting more than 8 weeks; 3) received a complete bronchial provocation test and standard treatment.

Exclusion criteria: 1) combined with other severe respiratory diseases, such as chronic obstructive pulmonary disease, bronchiectasis, etc.; 2) recent history of acute respiratory infection (within 3 months); 3) incomplete clinical data or missing key data.

### The procedure of bronchial provocation test

Informed consent was obtained from each patient prior to the bronchial provocation test. Jaeger Spirometer (MasterScreen, ‌CareFusion, Germany) was utilized for the test, with specific guidelines regarding the cessation of various medications prior to examination. The recommended time intervals for discontinuation were as follows: Short-acting β_2_ receptor agonists for 8 h, long-acting β_2_ receptor agonist for 48 h, short-acting theophylline for 12 h, medium and long-acting theophylline for 24–48 h, oral corticosteroids for 48 h, inhaled corticosteroids for 48 h, antihistamines for 72 h, leukotriene receptor antagonists for 96 h, and so on. Furthermore, on the testing day, patients were advised to refrain from consuming coffee, tea, and chocolate, as well as to avoid strenuous exercise or exposure to cold air for at least 4 hours. Airway hyperresponsiveness was measured using histamine diphosphate as an activator. Pulmonary function was measured after inhalation of each dose. The test was terminated when FEV_1_ and/or PEF decreased by ≥ 20% from the baseline value, or when the maximum cumulative dose of histamine diphosphate (2.4 mg) was reached, or when clinical discomfort was observed. Subsequently, 400 μg of salbutamol sulfate aerosol (Ventolin) was administered for inhalation. A decrease of ≥ 20% in FEV_1_ and/or PEF from the pre-provocation baseline was considered as the criterion for a positive bronchial provocation test [23].

### Model construction

Based on the collected data, a multivariate regression analysis method was employed to develop a mathematical model for predicting the probability of CVA diagnosis. Candidate variables included basic lung function indicators (such as FEV_1_/FVC, FEV_1_%pred, etc.), peripheral blood eosinophil count, FeNO, etc. Through stepwise regression analysis, variables that contributed significantly to predicting CVA were screened out, and the coefficients of each variable were calculated, ultimately, a nomogram model was constructed to predict the probability of CVA diagnosis.

### Model verification

In order to evaluate the accuracy and consistency of the constructed model, this study used the Bootstrap method to conduct internal validation of the nomogram, with the number of self-sampling B = 200.

### Statistical method

Data analysis was performed using SPSS (version 26.0) and R language (version 4.1.2). Continuous variables were described as mean ± SD or median (interquartile range), and categorical variables were expressed as frequencies and percentages. Deletion method was adopted for missing data, such as eosinophil count and FeNO value. Multivariate logistic regression analysis was used to construct the model, and the β coefficient, standard error, P value and 95% confidence interval of each variable were calculated. The accuracy of the predictive nomogram was evaluated using the predictive model receiver operating characteristic curve (ROC curve) and decision curve analysis (DCA). Additionally, the calibration curve of the prediction model was utilized for evaluating consistency. A *P* value of < 0.05 was considered indicative of statistical significance.

## Results

### Basic information of patients

**Fig. 1:**
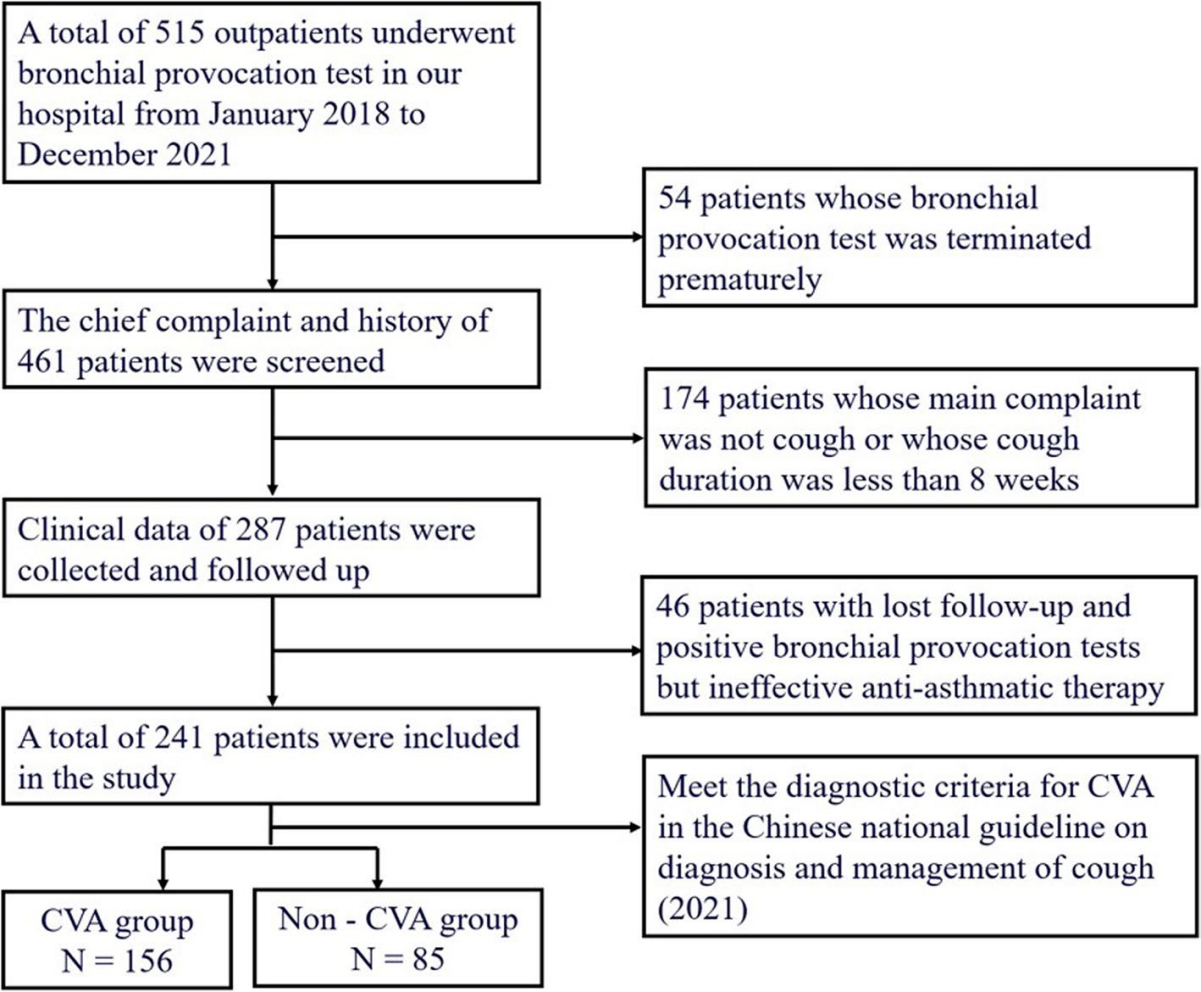
The flowchart of patients’ recruitment process

This study retrospectively analyzed the data of 515 patients who underwent bronchial provocation test at our hospital from January 2018 to December 2021. Based on the established inclusion and exclusion criteria, a total of 241 patients with chronic cough were ultimately included for analysis. This cohort comprised 156 cases of CVA and 85 patients with other types of chronic cough. The patients’ recruitment process was shown in Fig. 1.

### Results of univariate analysis

Univariate analysis was conducted on the collected variables using t-tests and χ^2^ tests. The results indicated that cough induced by cold air (χ^2^ = 16.001, *P* < 0.001) and cough induced by pungent odors (χ^2^ = 17.893, *P* < 0.001) were significant factors. Symptoms such as belching (χ^2^ = 8.099, *P* = 0.011), cough phase (χ^2^ = 26.390, *P* < 0.001), seasonal cough (χ^2^ = 11.034, *P* = 0.001), and chest tightness (χ^2^ = 20.106, *P* < 0.001) were also significant. Additionally, a history of allergic rhinitis (χ^2^ = 12.502, *P* < 0.001), family history of allergies (χ^2^ = 3.892, *P* = 0.049), and lung function indicators, such as FVC (t = -2.479, *P* = 0.014), FEV_1_%pred (t = -2.607, *P* = 0.010), PEF%pred (t = -4.520, *P* < 0.001), MEF50%pred (t = -4.166, *P* < 0.001), MEF25%pred (t = -2.591, *P* = 0.010), and the percentage of the predicted value of maximum mid-expiratory flow (MMEF%pred) (t = -2.985, *P* = 0.003), were found to be associated with CVA diagnosis (Tables 1 and 2).

**Table 1:** Clinical features of CVA and non-CVA patients

Clinical feature		CVA group	Non-CVA group	*χ*^*2*^/*Z*/*t*	*P*
Sex (n)	Male	61	44	3.588	0.058
	Female	95	41		
Age (year)		35 (28, 46)	36 (31, 42)	-0.250	0.803
BMI (kg/m^2^)		22.88 ± 3.08	23.15 ± 2.96	-0.668	0.505
Course of disease (week)		24 (12, 96)	48 (16, 120)	-0.936	0.349
Cough inducement (n)	cold air	30	1	16.001	< 0.001
	sport	7	0	2.498	0.114
	talking	4	0	0.924	0.337
	pungent odors	56	0	17.893	< 0.001
	special foods	9	0	3.616	0.057
	belching	14	0	8.099	0.011
	smoking	10	1	2.363	0.124
	keeping pets	4	0	0.924	0.337
Cough trait (n)	dry cough	143	73	1.980	0.159
	wet cough	13	12		
Cough phase (n)	Yes	76	13	26.390	< 0.001
	No	80	72		
Seasonal cough (n)	Yes	38	6	11.034	0.001
	No	118	79		
Chest tightness (n)	Yes	32	0	20.106	< 0.001
	No	124	85		
Chest pain (n)	Yes	3	0	0.460	0.497
	No	153	85		
History of allergic rhinitis (n)	Yes	43	7	12.502	< 0.001
	No	113	78		
Family history of allergies (n)	Yes	14	2	3.892	0.049
	No	142	83		

*CVA* cough variant asthma, *BMI* body mass index

**Table 2:** Lung function indexes in CVA and non-CVA patients

Lung function indexes	CVA group	Non-CVA group	*t*/*Z*	*P*
FVC (L)	3.75 ± 0.84	4.02 ± 0.74	-2.479	0.014
FEV_1_%pred (%)	98.08 ± 11.22	101.88 ± 10.02	-2.607	0.010
FEV_1_/FVC (%)	82.69 ± 6.98	82.95 ± 7.07	-0.282	0.778
FEV_1_/FVC%pred (%)	101.57 ± 8.33	100.62 ± 8.94	0.817	0.415
PEF%pred (%)	101.59 ± 15.09	111.04 ± 16.22	-4.520	< 0.001
MEF50%pred (%)	84.58 ± 23.43	98.25 ± 25.90	-4.166	< 0.001
MEF25%pred (%)	75.79 ± 26.22	85.29 ± 28.86	-2.591	0.010
MMEF%pred (%)	80.14 ± 22.63	89.27 ± 22.77	-2.985	0.003
FeNO (ppb)	20.00 (13.00, 34.00)	16.00 (11.75, 24.00)	-1.602	0.109

*FVC* forced vital capacity, *FEV*_*1*_ forced expiratory volume in 1 s, *PEF* peak expiratory flow, *MEF* maximum expiratory flow, *MMEF* maximum mid-expiratory flow, *FeNO* fractional exhaled nitric oxide

The blood routine test data of 76 patients were statistically analyzed. The results of eosinophil count in CVA group (*n* = 63) and Non-CVA group (*n* = 13) were 0.11 (0.05, 0.20) × 10^9^/L and 0.10 (0.07, 0.15) × 10^9^/L, respectively. The median serum total IgE in CVA group and Non-CVA group were (27.90 [9.55, 67.00] IU/L and 20.50 [4.00, 71.20] IU/L). In contrast, peripheral blood eosinophil count and serum total IgE levels were not identified as predictive factors for CVA (*P* = 0.772 and *P* = 0.827, respectively).

The FeNO values of 109 patients were statistically analyzed. The results of FeNO in CVA group (*n* = 79) and Non-CVA group (*n* = 30) were 20.00 ppb (13.00, 34.00) and 16.00 ppb (11.75, 24.00), respectively. FeNO was not identified as predictive factors for CVA.

### Multivariate logistic regression analysis

Correlation analysis and dimensionality reduction were conducted on the statistically significant factors identified above. Following this, multivariate logistic regression analysis was performed. The results indicated that cough induced by cold air (*P* = 0.019) and cough induced by pungent odors (*P* = 0.002), along with cough phase (*P* < 0.001), history of allergic rhinitis (*P* = 0.018), and MMEF%pred (*P* = 0.039), were independent predictors of CVA (Table 3).

**Table 3:** Multivariate regression analysis of CVA diagnosis and prediction

	ß	Standard deviation	Significance level	ExP (ß)	95%CI
Cold air as a contributing factor	2.525	1.076	0.019	12.493	1.517 ~ 102.886
Pungent odors	1.379	0.446	0.002	3.969	1.655 ~ 9.519
Cough phase	1.507	0.386	< 0.001	4.515	2.119 ~ 9.618
Seasonal cough	0.961	0.541	0.076	2.614	0.905 ~ 7.552
History of allergic rhinitis	1.173	0.496	0.018	3.231	1.223 ~ 8.536
Family history of allergies	0.848	0.858	0.323	2.336	0.434 ~ 12.566
FVC	-0.234	0.217	0.282	0.792	0.517 ~ 1.212
FEV_1_%pred	0.001	0.020	0.949	1.001	0.963 ~ 1.042
MMEF%pred	-0.020	0.009	0.039	0.981	0.963 ~ 0.999

*CVA* cough variant asthma, *FVC* forced vital capacity, *FEV*_*1*_ forced expiratory volume in 1 s, *MMEF* maximum mid-expiratory flow

### Construct a CVA diagnosis prediction nomogram model and verify its efficiency

**Fig. 2:**
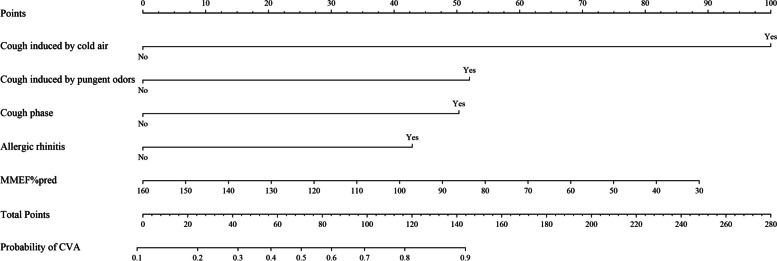
CVA diagnosis and prediction nomogram. The results of multivariate logistic regression analysis showed that cough induced by cold air (*P* = 0.019), cough induced by pungent odors (*P* = 0.002), along with cough phase (*P* < 0.001), history of allergic rhinitis (*P* = 0.018), and MMEF%pred (*P* = 0.039), were independent predictors of CVA. Based on the results of multivariate logistic regression analysis, a CVA diagnosis prediction nomogram model was developed

**Fig. 3:**
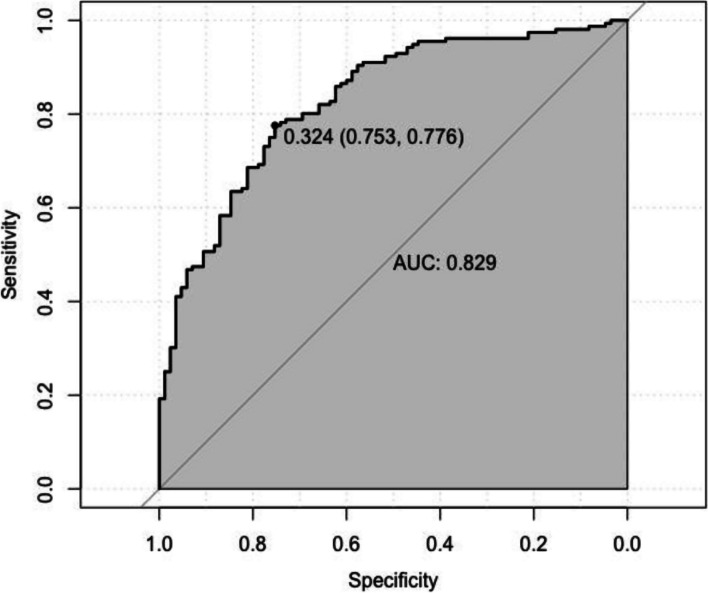
AUC of the CVA diagnostic prediction nomogram. This nomogram model had a high degree of discrimination. The area under the receiver operating characteristic curve (ROC) was 0.829, with a sensitivity of 75.3% and a specificity of 77.6%

**Fig. 4:**
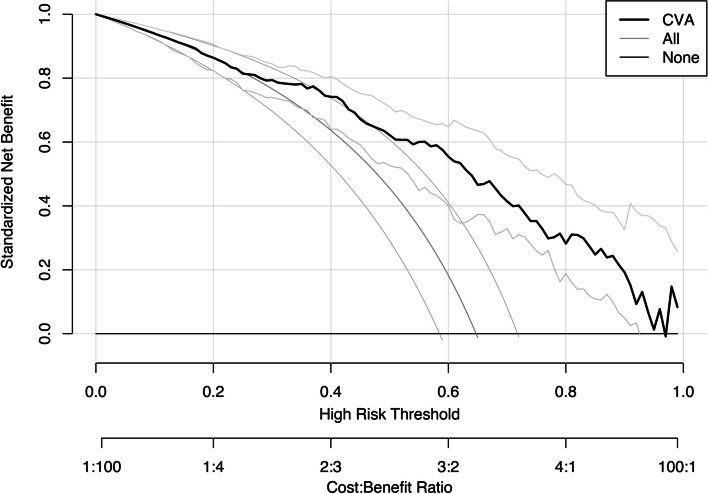
Decision curve of CVA diagnosis prediction nomogram

**Fig. 5:**
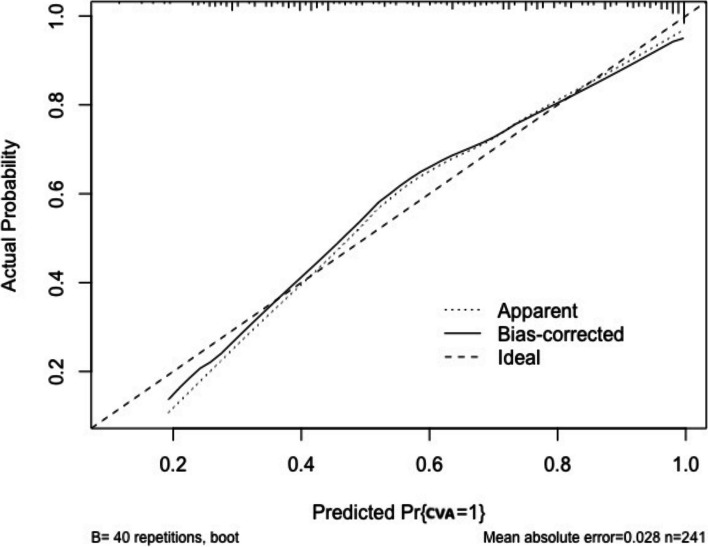
Correction curve of CVA diagnosis prediction nomogram

Based on the results of multivariate logistic regression analysis, a nomogram model for predicting a CVA diagnosis was developed (Fig. 2). The model underwent internal validation using the Bootstrap method, yielding a C-index of 0.920, which indicated a high degree of discrimination. Additionally, the area under the receiver operating characteristic curve (ROC) was 0.829, with a sensitivity of 75.3% and a specificity of 77.6% (Fig. 3). Further evaluation through decision curve analysis (DCA) confirmed the model's high accuracy (Fig. 4). Calibration curve analysis demonstrated good agreement between the predicted probabilities of the model and the actual probabilities (Fig. 5).

## Discussion

Chronic cough is a prevalent complaint among patients in general medicine and respiratory specialist outpatient clinics, with a global adult prevalence rate reaching as high as 9.6% [1–3]. This condition significantly impacts patients' quality of life and imposes a substantial socioeconomic burden. CVA is a primary cause of chronic cough in adults, making early diagnosis essential for timely treatment and for delaying the progression to typical asthma. The bronchial provocation test is regarded as a key method for diagnosing CVA due to its high sensitivity; however, its specificity is relatively low, and practical implementation is hindered by various factors, including the availability of testing equipment, patient acceptance, and contraindications [11, 12]. This challenge is particularly pronounced during public health events, such as the COVID-19 pandemic, which further restricts the use of bronchial provocation test [24, 25]. Consequently, the diagnosis of CVA presents challenges for patients unable to undergo the bronchial provocation test. Therefore, it is imperative to develop a CVA diagnostic prediction model that does not rely on bronchial provocation test.

This study included a total of 241 outpatients with chronic cough, of whom 156 were diagnosed with CVA, representing 64.7%. This prevalence is significantly higher than the 24% to 42% reported in previous studies. One possible explanation for this discrepancy is that clinicians may only refer patients for evaluation when there is a strong suspicion of CVA. Therefore, there was a certain selection bias, which might account for the elevated incidence of CVA observed in this study. A substantial amount of clinical data was collected, and both univariate and multivariate logistic regression analyses were employed to identify independent predictors of CVA diagnosis. These predictors included cold air-induced cough, pungent odors-induced cough, cough phase, a history of allergic rhinitis, and MMEF%pred. These factors are not only readily accessible in clinical practice but also have a clear pathophysiological basis. For instance, exposure to cold air can induce or exacerbate cough by activating airway neuroreceptors and causing bronchial smooth muscle contraction. Previous studies have indicated that certain cough triggers may be associated with specific underlying disorders [26–28]. Kanemitsu et al. studied 163 patients with persistent cough for over 3 weeks and identified cold air as the only cough trigger among the 18 examined that demonstrated a statistically significant difference between CVA and non-CVA patients [26]. They posited that cold air could serve as a cough trigger to aid in the diagnosis of CVA, particularly when FeNO levels were below 22 ppb. However, there remained some debate regarding the role of pungent odors as a predictive diagnostic indicator of CVA. The study by Kanemitsu et al. [26] concluded that there was no statistically significant difference between smoking or perfume odors in CVA patients compared to non-CVA patients. Our research results suggest that pungent odors induced cough is one of the indicators for predicting diagnosis of CVA, which aligns closely with the findings of Matsumoto et al. [27].

This study's model incorporated cough phasing, specifically cough occurrences in the early morning and/or at night. The timing of cough may be related to circadian rhythms. During sleep, the vagus nerve is activated, and the concentration of adrenocortical hormones decreases, leading to mild bronchoconstriction. Additionally, evidence suggests that bronchial hyperresponsiveness and airway resistance increase at night, resulting in nocturnal asthma symptoms [29, 30]. Our study indicated that CVA patients experienced significantly higher rates of morning and/or nighttime cough compared to non-CVA patients. This suggests that the cough pattern in CVA patients exhibits a specific phase, which aligns closely with findings from other studies. Furthermore, a history of allergic rhinitis, as an important host factor, is closely associated with allergic reaction-induced airway inflammation, characterized by increased eosinophil counts and elevated plasma IgE levels [31, 32]. This history serves as an independent predictive factor for the diagnosis of CVA.

In recent years, clinical studies have identified numerous biomarkers associated with asthma. Among the most representative clinical detection items in blood cell analysis are eosinophil count and serum total IgE. Elevated levels of these markers are linked to airway hyperresponsiveness in asthma patients and serve as important indicators for asthma diagnosis [33–35]. However, due to factors related to COVID-19 and other reasons, not all patients underwent routine blood tests, total serum IgE assessments, and FeNO testing. This study examined the blood cell profiles in 76 patients, assessed the total serum IgE levels in 48 patients, and tested the FeNO values in 109 patients. The findings indicated that the eosinophil count, eosinophil percentage, total serum IgE levels, and FeNO values in the CVA group were higher than those in the Non-CVA group. However, the difference between the two groups was not statistically significant. These results exhibited certain discrepancies when compared to previous literature reports [36–38]. Such differences may be attributed to the small sample size of this study and the varying degrees of inflammation present in CVA compared to typical asthma. Further studies with larger sample sizes are necessary to validate these findings.

Most studies suggested that the prevalence of asthma in adult female was higher than that in adult male until around menopause, then the prevalence of asthma in female began to decline after menopause [39]. However, in our study, female was not found to be an independent predictor of CVA. which may be attributed to several factors: (1) The incidence of asthma was related to BMI, the association was even stronger in female with a BMI > 28 kg/m^2^ [40, 41]. The BMI of female in our study was 22.88 ± 3.08, which was significantly lower than that reported in other studies. (2) In addition, the incidence of asthma varies among different ethnic groups. A survey in China found that the incidence was still higher in male than in female [42]. Therefore, whether the incidence of CVA is higher in female compared to male requires further confirmation through large-scale clinical studies.

The diagnosis of CVA should be based on the patient's symptoms, signs, bronchial provocation test, and other diagnostic results. In pulmonary function test, FEV_1_ and FVC reflect large airway function and are widely utilized to evaluate proximal airway obstruction. Small airways refer to peripheral bronchioles with an inner diameter of less than 2 mm, and damage to these small airways occurs in the early stages of various respiratory diseases [43, 44]. Numerous studies, both domestically and internationally, suggest that small airway lesions are associated with severe asthma [45, 46]. Recently, more and more researches have indicated that small airway lesions frequently manifest in mild asthma as well [47, 48]. The results of this study demonstrated that, compared to the Non-CVA group, lung function indicators, such as FVC, FEV_1_%pred, PEF%pred, MEF50%pred, MEF25%pred, and MMEF%pred were all significantly lower in the CVA group. This indicated that the airway functions of CVA patients were inferior to those of patients with non-CVA, aligning with conclusions drawn from previous literature [47]. A large-sample study revealed that MMEF%pred was a more sensitive indicator of small airway damage in asthmatic patients. Currently, the cutoff values used to evaluate small airway damage vary. In prior studies, an MMEF%pred of less than 65% has often been indicative of small airway disease [49, 50]. In this study, the average MMEF%pred of patients in the CVA group was 80.14 ± 22.63, which exceeded the commonly used threshold (< 65%). This discrepancy might be attributed to the fact that the primary study population in previous research consisted of typical asthma patients.

This study employed univariate regression analysis to identify that cold air and pungent odors induce cough, alongside factors such as the presence of acid reflux and belching symptoms, cough phase, cough seasonality, chest tightness, history of allergic rhinitis, family history of allergies, and various pulmonary function indexes (FVC, FEV_1_%pred, PEF%pred, MEF50%pred, MEF25%pred, and MMEF%pred) as relevant predictive factors for CVA diagnosis. Building on these findings, a multivariate regression analysis was conducted. The results confirmed that cold air and pungent odors were significant triggers for cough, while the cough phase, history of allergic rhinitis, and MMEF%pred emerged as independent predictors of CVA diagnosis. For the first time, a CVA diagnosis prediction nomogram model was constructed and validated. This model simplifies complex mathematical formulas into an intuitive chart format, facilitating quick calculations of a patient's CVA diagnosis probability for clinicians. Internal validation results indicate that the model exhibits good discrimination, consistency, and high predictive accuracy (AUC = 0.829, C-index = 0.920). While several scholars have developed prediction models for typical asthma diagnosis with favorable performance [18–22], no predictive nomogram model specifically targeting CVA has been previously reported. Consequently, the establishment of this model offers clinicians a new, convenient, and accurate diagnostic tool for patients unable to undergo bronchial provocation test, thereby aiding in the early diagnosis and intervention of CVA patients. However, the utility of this model in CVA diagnosis warrants further validation through subsequent studies.

This study has several limitations. Firstly, it was a single-center, retrospective analysis, and certain laboratory data and FeNO values were missing, which might introduce bias. Secondly, the study performed only internal validation and required prospective external validation in the future to assess the generalizability of the model. Thirdly, this nomogram included only individuals of the yellow race, and it remains to be determined whether the same findings apply to the white, black, and brown populations.. Lastly, the study did not incorporate long-term follow-up for the Non-CVA group, making it impossible to ascertain the long-term outcomes for these patients. Consequently, future studies will aim to expand the sample size, enhance data collection, and implement long-term follow-up to further validate and optimize the model. With the deepening research on the mechanism of CVA, the emergence of new biomarkers and advanced diagnostic techniques, including the development of artificial intelligence technology, will assist in further optimizing the nomogram's predictive power of CVA [51, 52].

## Conclusion

This study is the first to retrospectively analyze data from a cohort of patients with chronic cough who underwent bronchial provocation test. It employs univariate and multivariate regression analyses to establish associations with cold air-induced cough, pungent odors-induced cough, cough phase, history of allergic rhinitis, and maximum expiration. The MMEF%pred is identified as an independent predictor of CVA. Based on these indicators, a CVA diagnostic prediction nomogram model is constructed. Internal validation demonstrates that the model exhibits good sensitivity and specificity, with a strong correlation between predicted and actual diagnostic probabilities, indicating robust predictive performance. This model serves as a reliable diagnostic prediction tool for patients with chronic cough who are unable to undergo bronchial provocation test, highlighting its significant clinical application value.

## Data Availability

The datasets used and/or analysed during the current study are available from the corresponding author on reasonable request.

## Abbreviations

CVA: Cough variant asthma
ROC: Receiver operating characteristic curve
DCA: Decision curve analysis
BMI: Body mass index
FeNO: Fractional exhaled nitric oxide
FEV_1_: Forced expiratory volume in 1 s
FVC: Forced vital capacity
MMEF: Maximum mid-expiratory flow
MMEF%pred: The percentage of the predicted value of maximum mid-expiratory flow
PEF: Peak expiratory flow
MEF: Maximum expiratory flow
